# 46 Abdominal Wounds Treated in a Divisional Ambulance

**Published:** 1918-09

**Authors:** 


**46 Abdominal Wounds Treated in a Divisional Ambulance.** Seguninat, Le Prog. Med., Feb. 23. Operated 34 times with 12 cures. The interval before operation was 4½ hours or more.

